# Signs of Alzheimer’s Disease: Tied to Aging

**DOI:** 10.3390/ijms26114974

**Published:** 2025-05-22

**Authors:** Jiahui Chen, Zhongying Zhu, Yuanyuan Xu

**Affiliations:** Key Laboratory of Zoonosis Research, Ministry of Education, College of Animal Science, Jilin University, Changchun 130062, China; scb99051721@163.com (J.C.); zzy6459@163.com (Z.Z.)

**Keywords:** Alzheimer’s disease, amyloid cascade hypothesis, neuroinflammation, endothelial dysfunction, mitochondrial dysfunction animal models, therapies

## Abstract

**:** Alzheimer’s disease (AD) is a neurodegenerative disorder closely associated with aging, and its pathogenesis involves the interaction of multidimensional pathophysiologic processes. This review outlines the core mechanisms linking aging and AD. The amyloid cascade hypothesis emphasizes that abnormal deposition of amyloid-β (Aβ) triggers neuronal damage and synaptic dysfunction, which is exacerbated by aging-associated declines in protein clearance. Neuroinflammation, a synergistic pathogenetic factor in AD, is mediated by microglia activation, creating a vicious cycle with Aβ and tau pathology. The cholinergic hypothesis states that the degeneration of cholinergic neurons in the basal forebrain can lead to acetylcholine deficiency, which is directly associated with cognitive decline. Endothelial disorders promote neuroinflammation and metabolic waste accumulation through blood–brain barrier dysfunction and cerebral vascular abnormalities. In addition, glutamate-mediated excitotoxicity and mitochondrial dysfunction (e.g., oxidative stress and energy metabolism imbalance) further lead to neuronal death, and aging-associated declines in mitochondrial autophagy exacerbate such damage. This review also explores the application of animal models that mimic AD and aging in studying these mechanisms and summarizes therapeutic strategies targeting these pathways. Future studies need to integrate multi-targeted therapies and focus on the role of the aging microenvironment in regulating AD pathology in order to develop more effective early diagnosis and treatment options.

## 1. Introduction

Alzheimer’s disease (AD), also known as senile dementia, is the most common chronic neurodegenerative disease in the elderly. It is caused by widespread cerebral cortical atrophy and degenerative lesions and is mainly characterized by cognitive decline, as well as behavioral and personality disorders. Aging is the greatest risk factor for AD, accounting for more than 95% of cases [1]. It is a process in which the physiological integrity of an organism will gradually be lost over time, and the structure and function of tissues and organs will change, leading to the weakening of its adaptive ability to the internal and external environments, as well as the decline in survival ability [2].

Cellular senescence is considered to be a key marker of aging. In this process, cells will stop dividing and enter a state of permanent growth arrest. Senescent cells will accumulate in different tissues and organs and eventually have different physiological and pathological functions [3]. During the aging process, the body’s susceptibility to diseases increases significantly, and a variety of diseases occur frequently, including diabetes, cardiovascular diseases, musculoskeletal disorders, cancer, and neurological disorders. From a genetic or pharmacological perspective, eliminating senescent cells could extend the health span and lifespan of natural aging mice [4]. Through this method, various diseases with similar symptoms caused by aging could be treated [5].

**Figure 1:**
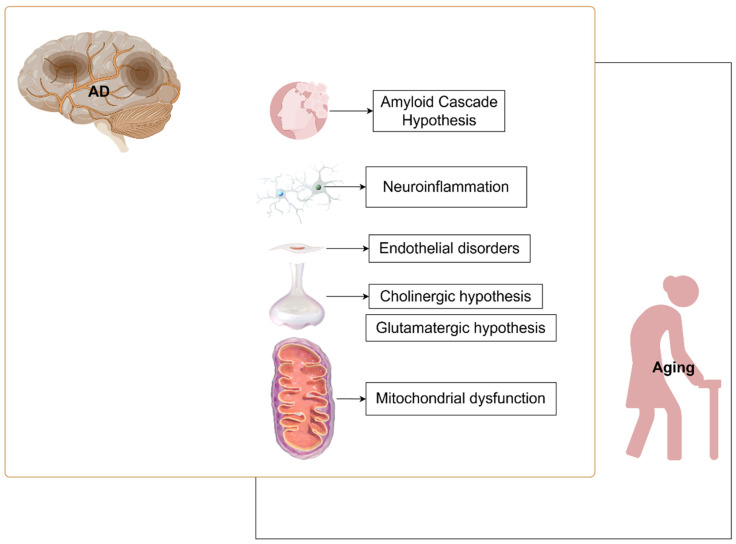
Common research areas between AD and aging. Amyloid cascade hypothesis, neuroinflammation, endothelial disorders, cholinergic hypothesis, glutamatergic hypothesis, and mitochondrial dysfunction. Neuroinflammation is mainly manifested in microglia and astrocytes; endothelial disorders describe the effects of the blood–brain barrier (BBB); the cholinergic and glutamatergic hypotheses express neuronal and synaptic effects; and mitochondrial disorders have many manifestations, such as metabolic disorders, mitochondrial autophagy, and others. This figure is created by FigDraw.

There is an extremely strong multidimensional connection between aging and AD. During aging, neurodegenerative changes, dysregulation of neurons (especially cholinergic neurons), and synaptic dysfunction are highly similar to the aggregation of β-amyloid (Aβ) and hyperphosphorylation of tau proteins in AD. In addition, the inflammatory response associated with aging provides a facilitating environment for the development of AD. Inflammatory factors will disrupt the blood–brain barrier and promote the aggregation of Aβ and the phosphorylation of tau proteins. Mitochondrial dysfunction also exists in both aging and AD [6,7,8,9,10]. In this review, we focus on the mechanisms connecting Alzheimer’s disease (AD) and aging (Figure 1), describing therapeutic strategies based on these connections. Additionally, we summarize some novel concepts that have emerged in recent years regarding AD, offering further perspectives into the research and development of diagnostic and treatment approaches. In view of the close and complex association between AD and aging, an in-depth study of the interactions between the two is conducive to exploring the pathogenesis of AD and developing more effective treatments. In this review, we will focus on the common influencing factors between AD and aging (as shown in Figure 1) and briefly overview the current research status of treatment.

## 2. Amyloid Cascade Hypothesis

Since the discovery of the amyloid precursor protein in 1987, it has triggered research into the molecular mechanisms of AD. Aβ and tau proteins have been intertwined in the pathogenesis of AD [11,12]. Various assays based on them are used in patients to observe the progression of AD. They are also found during the aging process.

### 2.1. Aβ

Aβ is thought to be involved in the pathogenesis of AD. In 1987, an amyloid precursor protein (APP) containing 695 amino acids was isolated from the cerebral cortex of AD patients [11]. It is mainly concentrated in the synapses of neurons and plays a key role in brain development, synaptic plasticity, and the brain’s intrinsic immune system [12]. After APP is produced by neurons, it can be decomposed by proteases. There are two main decomposition pathways: the α-decomposition pathway and the β-catabolic pathway. The production of Aβ is strictly dependent on the β-catabolic pathway, which is regulated by the enzyme β-secretase (BACE1) [13,14]. In a normal organism, β- amyloid is maintained at very low levels.

Aβ monomers are the individual molecules of Aβ, which further aggregate into clusters to form Aβ oligomers. Aβ oligomers are highly neurotoxic [15]. Researchers have proposed the amyloid cascade hypothesis, which states that β-amyloid deposition can lead to tau phosphorylation and tangle formation, in turn causing neuronal death. Meanwhile, tangles aggregate into neurotoxic amyloid plaques, leading to neuronal dysfunction and death [11,16]. Apolipoprotein E4 (APOE 4) is shown to be the largest genetic risk factor for late-onset AD, as it can impair Aβ clearance and promote Aβ accumulation in the brain [17]. In early-onset familial AD, human genetics indicates that mutations tend to cluster in the APP gene or presenilin (the catalytic subunit of γ-secretase). These mutations can modify the proteolytic processing of APP, leading to an increased Aβ42/Aβ40 ratio or enhancing the tendency for Aβ peptides to self-aggregate [18,19]. Meanwhile, post-translational modifications of key proteins during Aβ processing also play an important role in the pathogenesis of AD. Increased BACE1 SUMOylation has been demonstrated to be a significant factor in the progression of AD pathology [20].

Currently, detection of Aβ is focused on the reduction of Aβ42 in cerebrospinal fluid (CSF) and PET imaging of cerebral amyloid. In order to increase the generalizability of the assay, more minimally invasive and cost-effective blood-based biomarkers have been developed, and plasma biomarkers have also been developed for prediction at the individual level [21]. Based on some other research findings, researchers have reduced the emphasis on the direct influence of Aβ in the pathogenesis of AD and gradually shifted the focus to tau proteins [22].

### 2.2. Tau

Tau is a member of the microtubule-associated protein (MAP) group. It has been shown that tau is a typical “naturally unfolded” protein [23]. In normal neurons, tau proteins play important roles in microtubule stabilization, axonal transport, neuronal activity, ion transport, and neurogenesis. However, any deleterious alteration that triggers unusual folding and aggregation leads to the development of neurodegenerative diseases, collectively known as tau proteinopathies [24]. Hyperphosphorylation leads to the formation of hyperphosphorylated and self-aggregating tau (known as p-tau), which is a hallmark feature of various tau proteinopathies. In AD, more than 30 residues in tau are phosphorylated by various kinases, the most notable is GSK3β [25]. The hyperphosphorylated state of tau induces conformational and charge changes, leading to the presentation of microtubule-binding structural domains and the promotion of oligomerization and self-aggregation. Over time, aggregation of these tau forms neurogenic fiber tangles (NFTs) [26]. The truncated form of tau tends to fold and self-aggregate rapidly and immediately aligns to form oligomers, thereby increasing the propagation of amyloid toxicity [27,28].

Mutations in the tau gene can disrupt normal tau function. For example, the ΔK280 mutation can reduce the ability of tau to interact with microtubules, increase the tendency of tau to self-aggregate, and, therefore, promote the formation of PHF and NFT [29]. This mutation has been observed not only in AD, but also in other neurodegenerative diseases, such as chromosome 17-associated hereditary frontotemporal dementia and Parkinson’s disease (FTDP-17) [30]. The overexpression of ApoE4 in neurons, rather than in astrocytes, can increase tau phosphorylation in mice [31].

Phosphorylated tau protein (pTau181) levels have been used to differentiate plasma samples from control subjects and AD patients, while Tau and pTau181 can be measured in plasma to predict brain tau load and neurodegenerative changes [32,33]. Recently, plasma p-tau217 has been found to have excellent diagnostic performance for AD, especially in early-onset AD or atypical dementia, which is assessed in a specialized setting [34]. In addition, blood levels of a protein called MTBR-tau243 can accurately reflect the degree of tau protein aggregation in the brains of AD patients and correlate with the severity of disease progression [35].

### 2.3. AD Deteriorates with Aging

Myelin is a multilayered cell membrane structure wrapped around axons in the nervous system. Myelin structure deteriorates with age. In 2023, researchers found that myelin damage promoted Aβ production and reduced its clearance through two types of myelin dysfunction in mice. These mice showed typical symptoms of AD-like behavioral deficits [36]. This means that age-related myelin defects can directly or indirectly promote the formation of Aβ plaques. This study ties the relationship between aging and AD in a firm bond.

On the other hand, it was recently found that pathogenic soluble tau can be readily transmitted to primary astrocytes in vitro and in vivo, and pathogenic tau delivery can trigger phosphorylation of endogenous astrocyte tau, leading to microtubule cytoskeleton instability. Furthermore, pathogenic tau delivery to astrocytes effectively upregulated several markers of cellular senescence, demonstrating pathogenic tau-induced astrocyte senescence for the first time [37]. Aβ and Tau, recognized as the earliest important markers of AD, play a significant role in clinical diagnosis and reinforce the evidence of the additive effects of AD during aging.

## 3. Nerve Damage

The pathological process of AD is characterized by β-amyloid deposition and tau phosphorylation, which can lead to neuronal dysfunction when exceeding a certain threshold, resulting in neuronal dysfunction. This leads to a systemic collapse of neurological damage [37]. This cascade of events, involving neuroinflammation, vascular collapse, attenuation of cholinergic signalling, and synaptic deficits, culminates in a comprehensive decline in cognitive function.

### 3.1. Neuroinflammation

Neuroinflammation is triggered by astrocytes and microglia and is associated with amyloid and tau protein pathology. Microglia are brain-resident immune cells of the central nervous system, which are activated in response to threats and play an important role in clearing Aβ and maintaining the dynamic homeostasis of Aβ [38]. The current hypothesis is that in early AD, microglia are activated by Aβ and then microglia phagocytose Aβ. However, after a period of time, microglia become enlarged and are no longer able to process Aβ. Chronic activation of microglia can continuously trigger neuroinflammation, and then exacerbate neuronal damage and increase amyloid deposition through the production of pro-inflammatory cytokines (i.e., IL-1, TNF-α, IL-6) and toxic products, leading to neuronal cell death and increased amyloid deposition [39]. These cytokines, especially IL-6, can promote hyperphosphorylation of tau. Thus, gliotic proliferation is also associated with the regional distribution of NFT [40,41,42].

In addition, activated microglia can be divided into two types: M1 (pro-inflammatory) and M2 (reparative), which can switch phenotypes during AD [43]. Neuroinflammatory plaques are more abundant in the M2-dominant state, whereas NFT burden is higher in the M1 state [44]. Several studies have shown that microglia display two peaks of activation during the process of AD. One is during the early stages of AD, such as the preclinical and mild cognitive impairment (MCI) stages, and the other is during the progression of dementia, especially near the NFT [45]. However, whether their activation has a protective effect in response to amyloid deposition has not been fully elucidated.

Inflammatory plaques are also surrounded by reactive astrocytes [46]. Astrocytes can ensure the functional integrity of neurons and synapses and respond to injury. Microglia-induced IL-1 activates astrocytes, which mediate neuron and oligodendrocyte apoptosis. In individuals affected by AD, astrocyte degeneration and increased reactive astrocytes can be found. It is recognized that abnormal astrocyte proliferation can exacerbate the pathophysiology of AD [47]. Among these genetic factors, the APOE gene is also associated with neuroinflammation in microglia and astrocytes [48].

Positron emission tomography (PET) and translocator protein (TSPO) signals have long been used to detect cognitive domains and can certainly be used to detect cognitive abnormalities in AD. PET imaging is used to visualize and quantify molecular brain changes in patients, especially microglia activation and reactive astrocyte proliferation [49]. TSPO signal intensity can be co-localized with tau distribution in the brain [50]. It also correlates with the rate of cognitive decline and may serve as a biomarker to predict disease progression [51].

Furthermore, senescent cells can also be observed in the context of human age-related diseases, which are usually considered as part of the inflammatory response [52]. The old-associated secretory phenotype (SASP) is a fundamental feature of cellular senescence [53]. In AD, SASP occurs in different cell types, leading to the secretion of various pro-inflammatory cytokines. Researchers have found that exogenous NAD+ supplementation can attenuate microglia and astrocyte activation and reduce the release of pro-inflammatory cytokines [54]. In addition, immune senescence can lead to impaired immune function, thereby reducing the clearance of senescent cells, resulting in the accumulation of senescent cells, and further exacerbating inflammation [55,56]. Therefore, inflammation strengthens the link between AD and aging.

### 3.2. Endothelial Dysfunction

Numerous studies have shown that vascular damage is an important contributor to cognitive impairment, frequently occurring with aging in the elderly population [57]. Amyloid deposition and tau phosphorylation have been shown to exacerbate vascular damage [58,59]. Endothelial cells are a layer of cells on the inner wall of blood vessels with multiple important functions. They can regulate vascular tone, vascular smooth muscle cell (VSMC) proliferation, immune cell adhesion, and vascular inflammation through the production of biologically active factors in order to maintain vascular homeostasis [60,61,62]. Single-cell RNA-seq studies have revealed that endothelial cells are distributed in different vascular segments and brain regions. After analysis, it was found that they are functionally consistent with the artery wall-remodeling properties, the transport function of capillaries, and the sensitivity of veins to inflammatory signals [63,64]. Selective permeability imbalance caused by structural or functional abnormalities in the endothelial cell layer of blood vessels is called endothelial dysfunction.

The molecular mechanisms of endothelial dysfunction are complex, which involve the influence of multiple pathological stimuli, such as inflammatory factor-mediated processes and oxidative stress. Endothelial dysfunction manifests as damage of the blood–brain barrier (BBB) in brain tissues [65]. Disruption of the BBB is now recognized as an early indicator of neurodegenerative diseases. The collapse of BBB usually precedes dementia [66]. BBB dysfunction is also associated with AD. The co-occurrence of inflammation and cognitive impairment has been demonstrated in mouse models [67].

In AD patients, Aβ plaques are widely distributed in the brain parenchyma and within the cerebral blood walls, and the abnormal accumulation in cerebral blood walls can directly damage endothelial cells [68,69,70]. This will further increase the permeability of BBB, making it easier for Aβ and other harmful substances to enter brain tissue and trigger neuroinflammation [71]. BBB dysfunction can increase the permeability and leakage of peripheral inflammatory factors (e.g., TNF-α, IL-1β) into the brain parenchyma and activate microglia and release more pro-inflammatory factors, creating a positive feedback loop [72,73]. Therefore, inflammatory signals can further exacerbate neuroinflammation [74].

The damaging effects on endothelial cells include impaired energy metabolism and accelerated apoptosis. These effects are caused by oxidative stress, disrupting the structure of BBB and leading to vascular injury [75]. Oxidative stress increases the permeability of BBB to harmful substances and promotes neuroinflammation. This, in turn, has been shown to amplify oxidative damage through inflammatory cytokine release and glial activation [76,77]. Thus, damage of BBB caused by endothelial dysfunction further leads to neuroinflammation, oxidative stress, and neurotoxicity, exacerbating pathological deposits in the brain. Meanwhile, accompanying the aging process, endothelial cells gradually deteriorate, with morphological changes of the corresponding cells and a gradual loss of normal function. This will affect endothelium-dependent diastolic capacity, vascular permeability, and barrier dysfunction, and then trigger vascular leakage [78]. The above changes are caused by the impairment of vascular microcirculation, Aβ deposition, and leakage of pro-inflammatory factors [79]. Depending on the disease process, the degree of BBB damage can be reflected by soluble platelet-derived growth factor receptor-β in the blood [66]. Cell adhesion molecule-1 (VCAM-1) and intercellular adhesion molecule-1 (ICAM-1) have been used to assess vascular endothelial cell damage and inflammatory response and to detect the level of endothelial dysfunction, which can help to diagnose and monitor the progression of AD at an early stage [80].

### 3.3. Cholinergic Hypothesis

Cognitive decline, the main presenting symptom of AD, has long been associated with dysregulation of the cholinergic system. Cholinergic neurotransmission processes mainly occur in the forebrain, nucleus basalis meynert (NBM), and hippocampus, and they play a key role in various cognitive functions [81]. The central cholinergic nervous system can influence acetylcholine (ACh) levels by regulating ACh synthesis and release [82]. Acetylcholine (ACh), a key excitatory neurotransmitter involved in learning, memory, and other higher cognitive functions, is synthesized by the enzyme choline acetyltransferase (ChAT) [83]. The cholinergic hypothesis states that lesions of cholinergic neurons impair ACh production and affect neuronal communication, leading to memory deficits and other cognitive deficits [84,85]. This is because severe damage to cholinergic neurons in the NBM can result in a significant decrease in ChAT activity within the primary projection areas, namely the cerebral cortex and the hippocampus-regions associated with learning and memory. This has been observed in the cadavers of AD patients [86].

Furthermore, in the process of cholinergic neuron degeneration, the decline of cognitive ability and memory is accompanied by various other complications. Hypoglycemia can be detected even before the onset of AD symptoms [87]. Insulin resistance is observed in brain tissue affected by AD, particularly in the hippocampus and cerebral cortex. As a result of insulin resistance, AD patients with type II diabetes mellitus (T2DM) experience cognitive deterioration at twice the rate of AD patients without complications [88,89]. In this process, intracellular glucose availability is reduced, ACh synthesis is blocked, signal transduction is inhibited, tau hyperphosphorylation is activated, and oxidative stress is induced, further leading to cognitive decline [90]. Brain hypometabolism in AD patients can be detected using 18F-FDG PET, which measures the regional glucose consumption associated with the strength of glutamatergic synapses in local cerebral tissues [91,92]. It can show the characteristic patterns of AD neurodegeneration earlier than MRI in people with mild cognitive impairment [93].

### 3.4. Glutamatergic Hypothesis

Synaptic degeneration is one of the main causes of cognitive impairment. Glutamate is the most abundant excitatory neurotransmitter in the central nervous system (CNS) of mammals. It can be synthesized through various metabolic pathways and is involved in synaptic plasticity, learning, and memory formation [94,95,96,97]. The regulation of glutamate in the brain is thought to be controlled primarily through the glutamate/glutamine cycle, and excessive glutamate remaining after excitation can be taken up by astrocytes [98,99]. After depolarization of the presynaptic neuron, synaptic vesicles storing glutamate first fuse with the membrane and then release glutamate into the synapse. Subsequently, glutamate activates various ionotropic and metabotropic receptors on postsynaptic and presynaptic neurons, as well as glial cells [100,101]. In AD, impaired N-methyl-D-aspartic acid receptor (NMDA) receptor function is associated with abnormal synaptic gap glutamate concentrations, leading to synaptic loss, neuronal excitotoxicity and impairment, and neural network imbalance [96]. When glutamatergic signaling overactivates NMDA receptors, it stimulates excess Ca^2+^ to enter the mediator, leading to excitotoxicity [102]. Autopsy samples from the brains of AD patients clearly show the accumulation of Aβ oligomers in synapses and the loss of excitatory synapses in these regions [103,104].

Physiological aging is accompanied by decreased activity of glutamatergic neurons and reduced expression of postsynaptic NMDA receptors, leading to impaired synaptic plasticity and reduced neuronal excitability and synaptic transmission efficiency [105]. During aging, oxidative stress accumulation and neuroinflammation can further inhibit glutamate reuptake, generate excitatory neurotoxicity, and accelerate the onset of neurodegenerative disorders such as AD. Aβ and hyperphosphorylated tau proteins can further amplify this pathologic process by enhancing presynaptic glutamate release and interfering with NMDA receptor function [106]. CSF biomarkers such as NfL, neural granule protein (NG), synaptosome-associated protein 25, and visinin-like protein 1 can be used to assess axonal damage and synaptic dysfunction [107]. Recently, large-scale proteomic analyses have revealed that synaptic proteins are most strongly associated with cognitive impairment, and this correlation is independent of amyloid and tau proteins [108].

Aβ and hyperphosphorylated tau proteins can impair axonal transport, leading to disturbed vesicle release from glutamatergic presynaptic terminals, further disrupting neural network synchronisation. Combined with the description of cholinergic-induced dysfunction above, these effects gradually indicate that synaptic dysfunction is a central driver of AD, leading to dementia.

## 4. Mitochondrial Dysfunction

Mitochondrial state and function are markedly differentiated from the normal state in both the AD process and the aging process [9]. Mitochondrial dysfunction is closely related to both. This section focuses on the connection between mitochondrial dysfunction and metabolism, introducing the concept of ferroptosis into the pathogenic mechanism. It further amplifies the apoptotic cascade signaling to demonstrate the role of mitochondria in both physiological and pathological states.

### 4.1. Metabolic Dysregulation

Mitochondria are important organelles for calcium homeostasis and neuronal metabolism. In AD, mitochondrial energy metabolism is significantly impaired, manifested by the reduced activity of key enzymes in the tricarboxylic acid cycle (TCA) and abnormal function of the respiratory chain complexes [109]. Ca^2+^ activates the activity of these key enzymes and manifests itself as homeostasis dysregulation in AD. Therefore, it leads to a decrease in ATP synthesis [110]. In mouse models, significant hypometabolism of glucose and reduced expression of mitochondrial complex I–V were found in the parietal and insular regions of the cerebral cortex [111,112].

In the brain, approximately 90% of the oxygen-dependent ATP required for neurons to perform their functions is supplied by the mitochondrial electron transport chain (ETC), which undergoes oxidative phosphorylation. Thus, impairment of oxidative phosphorylation affects the CNS earlier than any other system [113]. Reduced activity of the ETC complexes in the cerebral mitochondria of the AD patients was found in postmortem brain specimens [114,115]. Both of these conditions can result in blockage of the electron transport chain and reduced ATP production. ApoE4, which has been mentioned many times before, can also interact with mitochondria, reducing the mitochondrial membrane potential and causing mitochondrial fragmentation, thereby impairing energy production and leading to AD [116,117]. In addition, the brain is the second-most lipid-rich organ in the human body, second only to adipose tissue [118]. Therefore, fatty acid metabolism in mitochondria is also an important source of energy metabolism. In AD, the activity of enzymes related to fatty acid metabolism is altered, leading to the blockage of oxidative processes and abnormal fatty-acid metabolism [119]. In conclusion, there is an inextricable relationship between mitochondrial dysfunction and metabolic disorders, both of which produce effects on AD.

There were many studies linking mitochondrial dysfunction to the aging process 20 years ago [120]. With age, the function of mitochondria gradually decreases, which is manifested by a decrease in the number, an increase in the size, and a decrease in the membrane potential. This loss of function leads to a decline in cellular energy metabolism, affecting the normal physiological function of cells and, thus, promoting the aging process [121]. Although mitochondria have self-protective mechanisms during the aging process, mitochondrial dysfunction can exacerbate aging when these mechanisms are insufficient to cope with injury. At the same time, mitochondrial DNA (mtDNA) mutations accumulate with age, leading to respiratory chain dysfunction and increased production of oxygen free radicals, which, in turn, will further accumulate mtDNA mutations, creating a vicious cycle [122].

### 4.2. Other Factors About Mitochondrial Homeostasis

In recent years, iron death has been found to play an important role in the pathologic process of AD. Iron imbalance leads to senile plaque (SP) deposition and NFT [123]. Excessive accumulation of ROS and intracellular lipid peroxides can induce iron death. High levels of free radicals (ROS/RNS) affect biomolecules and DNA and alter the expression of various stress-responsive genes, thereby impairing cell integrity and causing cell death [124,125]. Mitochondrial respiration and synthesis of nicotinamide adenine dinucleotide phosphate (NADPH) oxidase are the main sources of ROS [126,127,128,129,130]. Excess ROS acts synergistically with Aβ to enhance neuronal membrane permeability and promote calcium ion overload and mitochondrial membrane potential collapse [131]. Lipid peroxidation products also amplify the neuroinflammatory response by activating NLRP3 inflammatory vesicles in microglia, thus forming a vicious cycle [132]. In a study, Aβ inhibits complex IV function and promotes cytochrome C release by binding to ABAD (Aβ-binding alcohol dehydrogenase) on the mitochondrial membrane, triggering the apoptotic pathway [133].

In addition, mitophagy is an important component of mitochondrial function in normal physiological and biochemical exercise. Mitophagy is a key mechanism for the clearance of damaged mitochondria. Under physiological conditions, the PINK1/Parkin pathway triggers ubiquitylation and recruits autophagosomes to clear injured mitochondria by recognizing the breakdown of the mitochondrial membrane potential [134]. However, in AD patients, Aβ and tau proteins weaken the localization of PINK1 at the outer mitochondrial membrane, leading to the failure of Parkin recruitment and impaired autophagosome formation [135,136]. In addition, the disruption of the acidic environment and decreased tissue protease activity lead to abnormal lysosomal function and impaired autophagosome–lysosome fusion, resulting in the accumulation of damaged mitochondria and the release of more ROS [137]. Recent studies have pointed out that abnormal retention of stress granules in early AD can further exacerbate mitochondrial fragmentation and energy metabolism collapse by inhibiting mitochondrial autophagy-associated mRNAs (e.g., PINK1, Parkin) and suppressing their translation [138]. Together, these mechanisms lead to the accumulation of damaged mitochondria within neurons, driving the amplification of oxidative stress and apoptotic-signaling cascades.

In the context of aging, as time goes by, the accumulation of oxidative damage overwhelms cellular defensive mechanisms and ROS attacks mitochondrial components, leading to progressive tissue and organ decline and dysfunction. Lipid peroxidation, protein oxidation, and DNA damage directly disrupt cellular homeostasis, while impaired mitochondrial autophagy hinders damage repair [77]. These processes are tightly intertwined with the molecular features of aging (telomere depletion, epigenetic disorders), leading to impaired cell function, increased inflammation, DNA mutations, and systemic oxidative stress, further exacerbating the aging process and collectively contributing to the onset of neurodegenerative diseases.

## 5. Therapeutic Strategies for Aging and AD

Before entering clinical trials, therapeutic strategies must be validated in model animals. These models provide a manipulable experimental system for studying the mechanisms and treatments of human diseases by simulating physiological and pathological processes. In this review, we have compiled a list of commonly used aging animal models and AD animal models. Current therapeutic approaches primarily focus on anti-inflammatory and antioxidant strategies, as well as nerve damage repair. Additionally, precisely targeted interventions using gene editing technology, nerve repair through the differentiation potential of stem cells, and mitochondrial-targeted repair strategies are being explored. These efforts are complemented by scientifically guided exercise, healthy diet, and lifestyle. By integrating model animals with therapeutic strategies, final drug screening and efficacy validation can be effectively achieved.

### 5.1. Animal Models

#### 5.1.1. Animal Models of Aging

As a complex biological process, aging involves changes in multiple physiological functions. The construction of animal models that can simulate the characteristics of human aging is important for analyzing the molecular mechanisms related to aging, revealing the pathological basis of aging-related diseases, and screening intervention strategies for anti-aging and treating age-related diseases. Currently, common animal models of aging can be divided into three categories: natural aging models, induced accelerated aging models, and gene editing aging models.

Natural aging models can reflect the time-dependent characteristics of physiological aging but require a long experimental period [3]. Accelerated aging models can rapidly simulate aging phenotypes through oxidative stress or metabolic interventions and are suitable for drug screening. Gene-editing models focus on specific pathways (e.g., telomere depletion, DNA repair defects) and provide precise tools for mechanism studies [139].

In animal models of aging, central nervous system degeneration is highly correlated with mitochondrial dysfunction. In naturally aging mice, the accumulation of mtDNA mutations in brain tissue is directly associated with reduced complex I/IV activity, which can further disrupt energy metabolism and activate apoptotic pathways [140]. In D-galactose-induced mice, chronic oxidative stress leads to the activation of NLRP3 inflammatory vesicles, which can trigger microglial overactivation and the downregulation of synaptic protein expression [141]. In telomerase-deficient zebrafish, telomere depletion triggers impaired proliferation of neural stem cells, which can exacerbate cognitive decline [139]. These pathological phenotypes can be treated by targeting mitochondrial function, as described in the therapeutic section of this article.

#### 5.1.2. Animal Models of AD

Transgenic animal models of AD mimic core pathological features of AD by introducing human mutant genes (e.g., APP, PSEN1). Commonly used models include the APP transgenic model, Aβ transgenic model, APP/PS1 double transgenic model, and tau transgenic model. The APP transgenic mouse model, which is the most widely used model and initially constructed based on the transgenic expression of the human APP gene, can develop robust amyloid pathology with memory deficits [142,143,144,145,146,147]. The Aβ transgenic mouse model can develop amyloid pathology [148]. But there are currently no data on cognitive deficits. The APP/PS1 double transgenic mouse model, in which Aβ plaque deposition and neuroinflammation start at 6 months of age and are accompanied by spatial memory deficits at 12 months of age, is widely used for anti-Aβ drug evaluation [54].

Tau transgenic mice (e.g., JNPL3 and 3 × Tg mice) can mimic neuroprotectin fibril tangles by expressing mutant tau proteins to reveal the relationship between tau pathology and synapse loss [149,150,151]. These models promote the resolution of AD molecular mechanisms and the validation of intervention targets by accurately simulating Aβ deposition, abnormal tau phosphorylation, and neuroinflammation.

Hybrid models of aging and AD simulate the age-dependent AD deterioration process by integrating transgenic phenotypes with aging-related metabolic disorders. Generally, we can breed AD transgenic mice to old age, or induce accelerated senescence in such mice to obtain the hybrid model of senescence and AD. The aged APP/PS1 mouse model, SAMP8 rapid-aging mouse model, and D-galactose-induced aging-AD hybrid mouse model have been constructed [141,152]. By superimposing aging and AD phenotypes, these models elucidate how age-related metabolism, inflammation, and mitochondrial dysfunction synergistically accelerate neurodegeneration and provide a theoretical basis for the development of intervention strategies to delay the dual phenotypes. These models mentioned above are summarized in Table 1.

### 5.2. Therapeutic Strategies

#### 5.2.1. Preclinical Strategies

In recent years, gene-editing technologies have emerged, demonstrating significant advancements in accuracy, stability, and applicability. These technologies hold great promise for gene therapy related to aging and AD [172]. Meanwhile, stem cells have strong differentiation ability, and different types of stem cells show different advantages in repairing nerve injury. Preclinical therapies enable restorative treatments through precise gene-editing techniques and stem cells with enhanced differentiation capabilities.

In targeting the APOE gene, the strong genetic risk factor for AD, gene-editing technology can reduce the genetic risk of AD by converting APOE4 to APOE3 [173]. By editing the APP gene or repairing progerin (PSEN1/PSEN2) mutations, the production of toxic Aβ is reduced [174]. Preclinical studies have validated the effectiveness of these strategies in mouse models. Knocking out the BACE1 gene can reduce Aβ plaques and improve cognitive function [175]. CRISPR technology can also be used to interfere with aging-related genes to slow or reverse AD progression. Genome-wide CRISPR screening revealed that the inhibition of the neddylation pathway can accelerate neuronal senescence and exacerbate AD neurodegeneration, suggesting that this pathway may be a potential target for regulating AD progression [176]. Additionally, autophagy is impaired in senescence and AD, resulting in the aggregation of aberrant proteins. Related genes (e.g., TFEB) can be repaired by CRISPR technology to enhance cellular clearance [177]. CRISPR technology also provides a tool for resolving multigene networks. Screening revealed that modulation of the co-expression network of genes such as CD48/CD40 can ease the condition [178]. These findings provide a theoretical basis for multi-targeted combined interventions.

Neural stem cells (NSCs) have the ability to differentiate into neurons, astrocytes, and oligodendrocytes, which can replace lost neurons in AD due to Aβ deposition and tau protein hyperphosphorylation and are suitable for repairing hippocampal and cortical neuronal damage in AD [179,180]. Moreover, NSCs also inhibit neuroinflammation [181]. Spatial transcriptomics studies have demonstrated that transplanted NSCs can modulate the expression of genes related to synaptic plasticity and metabolism and promote the reconstruction of neural circuits in the hippocampal region [182]. In addition, the transplantation of NSCs, in combination with a cholinesterase inhibitor, significantly improves cognitive function in a 5 × FAD mouse model, suggesting that the combination therapy enhances the efficacy of treatment [183].

Other types of stem cells also demonstrate distinct advantages in repairing nerve damage. Induced pluripotent stem cells (iPSCs) can be guided to differentiate into specific neuronal subtypes (e.g., cholinergic neurons), which are used to accurately replace lost cells in AD [184]. In addition, adipose-derived stem cells can secrete enkephalinase (Neprilysin), which can directly degrade Aβ plaques [185]. The transplantation of bone marrow mesenchymal stem cells (BMMSCs) ameliorates memory impairment in the AD mouse model through activation of the Wnt/β-catenin pathway [186]. The phase 2a study results of laromestrocel, the first mesenchymal stem cell (MSC) therapy to enter the clinical stage of AD, were recently published. The findings indicate that both single and multiple doses of laromestrocel, in patients with mild AD, can help reduce the rate of brain atrophy and address underlying cognitive decline, all with a favorable safety profile [187]. Compared to lecanemab, aducanumab, and donanemab, the therapy exhibited a superior safety profile. Although the primary objective of the study was to focus on safety, exploratory analysis revealed a trend towards a slower rate of cognitive decline in the laromestrocel treatment group. In the future, larger phase 3 clinical trials could be conducted to confirm both the long-term safety and the significance of the cognitive protective effect [188]. Thus, stem cell therapy provides a new direction for AD treatment. However, issues such as low post-transplantation survival rates, long-term safety, and standardized treatment processes still need to be addressed.

#### 5.2.2. Interventions

Neuroinflammation is a key factor in accelerating disease and aging progression, as well as a key to therapeutic interventions. Moreover, mitochondrial dysfunction is a prominent feature of aging and AD. By reducing neuroinflammation and enhancing mitochondrial function, the disease process can be effectively intervened.

Modulation of the myeloid triggering receptor 2 (TREM2)-signaling pathway and the vascular endothelial growth factor (VEGF)-signaling pathway can improve neuroinflammation by inhibiting microglia overactivation and maintaining blood–brain barrier integrity [189,190]. A recent study by Brandao et al. reported that xenon (Xe) can reduce amyloidosis and inflammation and protect against microglia activation in AD. This was demonstrated by the mouse model 5xFAD and APP/PS1 [191]. The natural product ginkgolide B (GB) can modulate the phenotype of microglia (M1 to M2 polarization) and inhibit the NLRP3 pathway to reduce the Aβ-induced inflammatory response [192]. NLRP3 inflammatory vesicles are a core component of the innate immune system in microglia. The sustained activation of NLRP3 not only promotes the deposition of Aβ and tau but also disrupts the blood–brain barrier by releasing inflammatory factors and attracting peripheral immune cells infiltration, resulting in a self-amplifying inflammatory cycle [193]. Compounds such as NLRP3-IN-2 and JC124 can block the interaction of NLRP3 with ASC and inhibit the inflammatory vesicles [194]. Some Chinese herbal compounds (e.g., bovine cerebroside and iguanodine) can exert synergistic anti-inflammatory effects through simultaneous inhibition of the NLRP3, NF-κB, and MAPK pathways [195]. In addition, supplementation with folic acid or betaine can enhance the metabolic buffering capacity and alleviate inflammation in AD [196].

Recent studies have shown that the genetic ablation of IL-12 signaling can reverse the loss of mature oligodendrocytes, restore myelin homeostasis, and rescue the amyloid-dependent reduction in β-protein-positive interneurons. This finding indicates that neuroinflammation can not only serve as an intervention for AD but also as a viable therapeutic target for this disease [197].

To realize the therapeutic potential of targeting mitochondria, intervention strategies focusing on antioxidants and their dynamic homeostasis have been explored from the perspective of mitochondrial biological functions and mechanisms. To date, a significant number of therapeutic approaches and pharmacological studies have been conducted using mouse models.

In terms of antioxidants, studies have shown that CoQ10 can help alleviate oxidative damage caused by mitochondrial dysfunction in AD, but its clinical efficacy still needs to be further verified. Idebenone, as a CoQ10 analog, can effectively scavenge a variety of free radicals [198]. NAC (N-acetylcysteine), on the other hand, can neutralize free radicals and repair oxidative damage by elevating intracellular glutathione (GSH) levels. It has been shown that NAC can significantly improve mitochondrial complex activity and gene expression and promote the recovery of mitochondrial function in a rat model of diabetes [199]. Thus, in AD, NAC has the potential to alleviate mitochondrial oxidative stress and impaired energy metabolism through similar mechanisms. This is also the case for PPARγ/PGC-1α agonists (e.g., pioglitazone) [200]. Additionally, SS-3 is a mitochondrial-targeted antioxidant that can reduce oxidative stress in mitochondria, thereby reducing neuronal damage [201].

Apelin-13 can exert neuroprotective effects through the PPARγ/PGC-1α-signaling pathway and has the potential to activate mitochondrial biosynthesis [202]. In addition to enhancing mitochondrial biosynthesis, enhancing mitochondrial autophagy is also an aspect. Two substances, urolithin A and spermidine, can activate the PINK1/Parkin-dependent mitochondrial autophagy pathway to clear damaged mitochondria. PPARγ activation can be used to inhibit iron death and delay AD through this pathway [203]. Mdivi-1 is a mitochondrial division inhibitor. In AD models, mitochondrial division/fusion imbalance is closely related to neuronal injury, and the application of Mdivi-1 can reduce mitochondrial fragmentation and improve neuronal survival [204]. S89 can specifically activate the mitochondrial fusion protein, MFN1, and promote mitochondrial fusion [205]. Blarcamesine, an oral small molecule agonist targeting the Sigma-1 receptor, is effective in improving the level of mitochondrial activation and ameliorating neuroinflammation in neuronal cells [206]. Currently, Blarcamesine has completed Phase 2a and Phase 2b/3 clinical trials for AD [207,208]. In addition, Ebselen can inhibit the formation of mitochondrial permeability transition pore (mPTP), thereby improving mitochondrial function. Ebselen can also improve mitochondrial biocompetence, synaptic function, and learning memory, as well as inhibit neuroinflammation in an AD mouse model [209].

In the clinical process, anti-inflammatory measures and improvements in mitochondrial function are employed to intervene in disease progression and slow down its deterioration. However, these intervention strategies only target one aspect of AD and cannot serve as the sole backbone of its treatment.

#### 5.2.3. Prevention

The role of exercise in promoting health and longevity has long been widely recognized. In addition to exercise, a daily diet is an important factor. To achieve optimal prevention of AD, it is recommended to pay attention to both exercise and dietary structure.

Appropriate exercise can enhance cognitive function, improve memory, and help prevent AD [210]. A meta-analysis noted that exercise can reduce the risk of dementia and AD by 28% and 45%, respectively, and higher levels of daily exercise are associated with a lower risk of AD [211]. Exercise can explicitly improve or maintain physical fitness in terms of aerobic capacity, muscle strength and endurance, as well as balance, coordination, and flexibility [212,213]. Appropriate and moderate amounts of exercise can help delay the onset and progression of AD.

Epidemiologic studies have provided evidence for the relationship between dietary patterns and AD. Relevant studies have shown that excessive intake of saturated fat (SF), simple carbohydrates, and high glycemic index foods is associated with an increased risk of AD onset [214]. Low-circulating choline is associated with the neuropathologic progression of AD, illustrating the importance of adequate dietary choline intake in counteracting the disease [215]. The gastrointestinal tract is colonized by a range of microorganisms, which are referred to as the “gut microbiota” [216,217]. According to recent studies, an increase in Actinobacteria and Ascomycetes, and a significant decrease in thick-walled bacteria and bifidobacteria, have been observed with age [218,219]. That is to say, in addition to their own roles, some nutrients in the food also have an impact on the structure, function, and secretion of metabolites in the intestinal flora. More and more research is focused on elucidating the bidirectional communication pathway between gut bacteria and the CNS, the microbiota–gut–brain axis [220]. Perhaps, in the future, it may become the root of a major treatment for AD or aging.

Overall, the therapeutic strategy for AD is based on genetic and stem cell research, supplemented by anti-inflammatory and antioxidant studies, while also incorporating a healthy lifestyle. This approach aims to maximize interdisciplinary and multifaceted synergies for the effective treatment of the disease.

## 6. New Perspectives on AD

**Figure 2:**
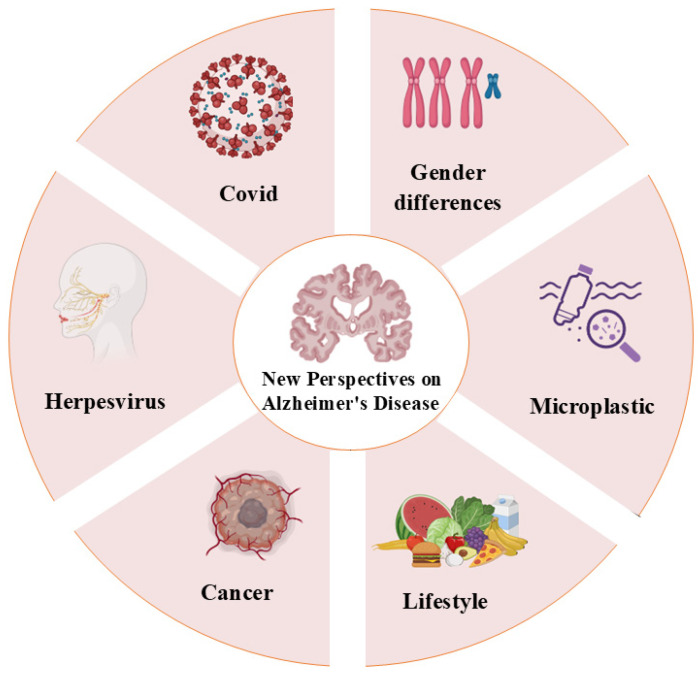
In recent years, new perspectives on AD include COVID, herpesvirus, cancer, lifestyle, microplastic, and gender differences. Dietary structure and physical activity are the main influencing factors of lifestyle, while gender differences are reflected in the susceptibility of females to AD. The material comes from biorender.

In recent years, many new perspectives on the factors affecting AD have emerged (Figure 2), focusing on susceptibility to induced disease and new fields. Since the 2020 COVID-19 pandemic, researchers have found that people infected with COVID-19 are more susceptible to AD, and this year, changes in plasma levels were also found as evidence [221]. In addition to COVID-19, it has also been found to have a strong association with herpesvirus [222].

In terms of gender differences, women are more susceptible to the disease than men. Some studies have shown that one of the main disparities is the level of plasma-free carnitine. This level of reduction may be responsible for women being more susceptible to neurodegenerative diseases such as AD [223]. In addition, this study implicates that plasma LAC and free carnitine may be considered as valid biomarkers for the early diagnosis of AD. Ma et al. found that, in cancer survivors, the probability of developing AD was significantly lower than that in normal individuals, and the reduction rate varied across cancers. This finding suggests that cancer patients have a more stable cognitive state [224]. Recently, scientists have creatively discovered the presence of microplastics in the body, and the accumulation in the brain is significantly higher than in other tissues, especially in AD patients [225]. This reveals that plastics/nanoplastics have an impact on human health.

## 7. Conclusions and Future Prospects

AD, as a neurodegenerative disease, is inextricably linked to aging. They share a complex molecular network and a highly intertwined pathological process. In the context of aging, aberrant aggregation of Aβ and Tau is amplified, and aging increases susceptibility to neuroinflammation, as well as decreases synaptic plasticity. Mitochondria may serve as the critical link between aging and AD, and their dysfunction can lead to an energy crisis, exacerbating protein misfolding and neuronal death. Aging is not only a risk factor for AD but also a “catalyst” for its pathological cascade.

People with AD not only pose challenges to themselves, but also to their families. AD hinders personal development and gradually has a lasting negative effect on societal function. While exploring potential therapies, animal models exhibit certain limitations, as they cannot fully replicate the complexity of human aging. Future studies should integrate multi-omics techniques and promote interdisciplinary collaborations to bridge the gap between mechanism understanding and clinical application.

We should investigate the common pathways between aging and AD to identify intervention targets that address multiple issues simultaneously. We can promote lifestyle interventions that emphasize better dietary management and exercise routines. Additionally, leveraging the current advancements in artificial intelligence can help integrate multi-omics data, establish AD risk-prediction models, and incorporate AD research into a broader framework of “health and aging”. By incorporating AD research into the “Healthy Aging” paradigm, we can share insights on anti-aging with fields like cancer and metabolic diseases.

## Figures and Tables

**Table 1:** Animal models related to aging and AD.

Model	Animals	Method	Refs.
Natural aging models	Nematode worm	Natural growth to senescence stage; typical lifespan: 1 month.	[153]
	Fruit fly	Natural growth to senescence; typical lifespan: 3 months.	[154]
	Danio rerio	Natural growth to senescence; typical life span: 36–42 months.	[155]
	Mice	Natural growth to senescence; typical life span: 2–4 years.	[144,156,157]
Accelerated-aging model	Mice	Rapidly aging (SAMP) mice; rapidly developing aging characteristics after 4–6 months of age.	[152]
	Rats	D-galactose induction	[158,159]
Gene editing models of aging	African killifish (Medaka)	Breeding with gene editing reduces lifespan to 9–26 weeks.	[160]
	Danio rerio	Telomerase-deficient; median life expectancy reduced to 9 months.	[139]
	Mice	Rps9 D95N mutation	[161]
		Hyperactivation of the tumor suppressor P53.	[162]
		INK-ATTAC type (knockout P16)	[163]
		PolgA gene proofreading functionally defective phenotype.	[164]
hAPP transgenic AD model	Mice	PDAPP type (V717F mutation introduced in APP)	[142]
		Tg2576. (Introduction of the K670N/M671L mutation in APP)	[143,144]
		APP23 type. (K670N/M671L and V717I double mutation in the APP gene)	[145]
		TgCRND8 type (K670N/M671L and V717I double mutation in APP gene)	[146,147]
Aβ transgenic AD model	Mice	BRI-Aβ42 Type A (BRI protein was used as a carrier to fuse Aβ42 to its C-terminus)	[148]
APP/precocene double-turn AD model	Mice	PSAPP type (Tg2576 × PSI)	[165]
		APPswe/PS1 ΔE9 type. (synergistic expression of APP with the K670N/M671L double-site mutation and deletion of exon 9 of PSEN1 (ΔE9))	[54,166,167]
		5 × FAD type (APP triple mutation and PSEN1 double mutation hybridization)	[168,169]
		2 × KI type (APP and PSEN1 double knock-in mutation)	[170,171]
hTau transgenic AD model	Mice	JNPL3 type (introduction of the tau gene carrying the P301L mutation)	[149]
		3 × Tg type (Integration of APP double mutation, single mutation in PSEN1 gene, triple mutation in Tau gene P301L)	[150]
		TAPP type (Tg2576 × JNPL3)	[151]
Aging-AD hybrid model	Mice	D-galactose induction	[141]
